# Brain Morphometric Changes Associated With Childhood-Onset Systemic Lupus Erythematosus and Neurocognitive Deficit

**DOI:** 10.1002/art.38009

**Published:** 2013-07-26

**Authors:** Darren R Gitelman, Marisa S Klein-Gitelman, Jun Ying, Anna Carmela P Sagcal-Gironella, Frank Zelko, Dean W Beebe, Mark DiFrancesco, Todd Parrish, Jessica Hummel, Travis Beckwith, Hermine I Brunner

**Affiliations:** 1 Northwestern University, Feinberg School of MedicineChicago, Illinois; 2 Ann & Robert H. Lurie Children#x0027;s Hospital of Chicago and Northwestern University, Feinberg School of MedicineChicago, Illinois; 3 University of Cincinnati College of MedicineCincinnati, Ohio; 4 Cincinnati Children#x0027;s Hospital Medical Center and University of Cincinnati College of MedicineCincinnati, Ohio

## Abstract

**Objective**To use structural magnetic resonance imaging (MRI) to characterize changes in gray matter and white matter volumes between patients with childhood-onset systemic lupus erythematosus (SLE) and matched controls, between patients with childhood-onset SLE with and those without neurocognitive deficit, and in relation to disease duration and treatment with steroids.

**Methods**Twenty-two patients with childhood-onset SLE and 19 healthy controls underwent high-resolution structural MRI. Probability density maps for gray matter and white matter were compared between groups.

**Results**Neuropsychological testing confirmed the presence of neurocognitive deficit in 8 patients with childhood-onset SLE. Multiple brain regions had reduced gray matter volume in the patients with childhood- onset SLE with neurocognitive deficit versus controls or patients with childhood-onset SLE without neurocognitive deficit. Neither disease duration nor cumulative oral or intravenous steroid doses accounted for decreases in gray matter. White matter volume was also reduced in patients with childhood-onset SLE with neurocognitive deficit, and the reduction was positively associated with both disease duration and cumulative oral steroid dose. Conversely, higher cumulative intravenous steroid doses were associated with higher white matter volumes.

**Conclusion**Neurocognitive deficit in patients with childhood-onset SLE is associated with multifocal decreases in both gray and white matter volumes. Since only white matter volume changes are related to disease duration and cumulative oral steroid use, this may suggest that gray and white matter alterations relate to different underlying mechanisms. Further work is needed to understand the relationship between gray and white matter alterations in childhood-onset SLE, whether the underlying mechanisms relate to immunologic, vascular, or other causes, and whether the changes are reversible or preventable. Likewise, the protective properties of intravenous steroids in maintaining white matter volumes require confirmation in larger cohorts.

Systemic lupus erythematosus (SLE) is the prototypical autoimmune disease, characterized by the presence of autoantibodies directed against multiple organ systems. SLE is diagnosed in ∼10% of patients during childhood (childhood-onset SLE). A proposed risk factor for poor prognosis is the presence of central nervous system involvement (1–2). Since publication of the new American College of Rheumatology (ACR) case definitions for neuropsychiatric SLE (NPSLE) (3), studies have confirmed that NPSLE is present in as many as 80% of adults with SLE (4–5), with neurocognitive deficit observed in 50–80% of these patients (4–6). Neurocognitive deficit often involves attention, visuoconstructional ability, and working memory (7). NPSLE may be even more common in children than among adults with SLE, with up to 95% of pediatric patients manifesting at least one symptom of NPSLE (5). Headache is the most common manifestation in children with NPSLE, while neurocognitive deficit has been described in 43–59% of patients (5–8).

In both children and adults the etiology of NPSLE is unknown. Standard clinical magnetic resonance imaging (MRI) techniques using measures of ventricular size, the number of T2 hyperintense lesions, or global measures of tissue integrity (9–10) have shown a relationship to neurocognitive deficit in adults with SLE. Studies of childhood-onset SLE suggest imaging abnormalities in 20–46% of patients, as determined using qualitative or subjective measures of lesion burden and measures of global atrophy (11,12). However, such measures may lead to bias due to the subjective and operator-dependent nature of the assessments, and are unable to localize specific regional involvement in childhood-onset SLE–associated neurocognitive deficit. Some recent imaging studies of adults with SLE, using advanced computational techniques (14,15), have demonstrated a variety of regional brain abnormalities associated with neurocognitive deficit and have shown relationships between brain alterations, disease duration, and steroid use (14,15).

Among these advanced techniques is voxel-based morphometry, which uses semiautomated algorithms to quantify gray matter and white matter volumes on a voxel-by-voxel basis across the entire brain. Hence, the technique is not biased by the selection of specific brain regions of interest, and, importantly, is independent of operator skill (17).

Voxel-based morphometry has not yet been applied to patients with childhood-onset SLE to explore and quantify imaging correlates of neurocognitive deficit. The objective of this study was to use voxel-based morphometry and high-resolution structural MRI to characterize gray matter and white matter volumetric differences between patients with childhood-onset SLE and matched controls, the relationship of childhood-onset SLE–associated neurocognitive deficit and gray matter and white matter volumes, and associations of disease duration and steroid treatment with gray matter and white matter volumes.

## PATIENTS AND METHODS

### Study design

Between March 2009 and May 2011, a subset of subjects participating in a larger longitudinal study (18) of childhood-onset SLE–associated neurocognitive deficit conducted at 2 sites (Cincinnati and Chicago) were asked to undergo MR scanning. Enrollment in the cross-sectional MRI substudy was determined by a lack of contraindications to MRI, including braces or claustrophobia. An attempt was also made to recruit equal numbers of patients with childhood-onset SLE with and without neurocognitive deficit, although balancing of the study population could not be achieved by the time recruitment was closed. The study was approved by the Institutional Review Boards at Cincinnati Children#x0027;s Hospital, Ann & Robert H. Lurie Children#x0027;s Hospital of Chicago, and Northwestern University.

### Patients with childhood-onset SLE

Index patients fulfilled the ACR classification criteria for SLE (19) prior to age 16 years and were between the ages of 9 and 18 years at the time of the study. Excluded from participation were patients with childhood-onset SLE with a history of any comorbid conditions affecting neurocognitive functioning that preceded the diagnosis of childhood-onset SLE, including neuropathy, movement disorders, seizures, central nervous thrombosis, or the presence of known structural brain abnormalities. Eligible patients were recruited consecutively into the study, their medical records were reviewed, and disease activity and damage were assessed at the time of the study (18). Specifically, we recorded disease duration and steroid exposure, the latter as a surrogate marker of treatment intensity.

### Controls

Each index patient with childhood-onset SLE (and/or the patient#x0027;s legal guardian) was asked to identify a healthy control. Besides the absence of known structural brain abnormalities, a control had to be within 1 year of the age, in the same school grade, and of the same sex as the index patient with childhood-onset SLE. Such a matching approach is commonly used to control for demographic, environmental, or socioeconomic factors when comparing groups of subjects for certain disease outcomes, including neurocognitive functioning (20). Similar to childhood-onset SLE patients, controls provided sociodemographic information and underwent a physical examination, with additional details provided elsewhere (18).

### Formal neuropsychological testing

The diagnosis of neurocognitive deficit required the administration of a standardized set of neuropsychological tests (21). All participants underwent the same 3-hour formal neuropsychological testing performed by a trained psychometrician, using the standardized neuropsychological battery for childhood-onset SLE (21). Briefly, the following tests were administered: the Wechsler Abbreviated Scale of Intelligence (WASI) Vocabulary, Block Design, and Matrix Reasoning subtests (22); the Wechsler Intelligence Scale for Children IV (WISC IV) Digit Span, Letter-Number Sequencing, Coding and Symbol Search subtests (23–24); the Woodcock-Johnson III achievement subtests (Letter-Word Identification, Reading Fluency, Calculations, and Math Fluency) (25); the Wide Range Assessment of Memory and Learning, Second Edition screening battery, consisting of the Story Memory, Verbal Learning, Design Memory, and Picture Memory subtests (26); the Inhibition versus Color Naming Contrast Score of the Delis-Kaplan Executive Function System (27); Conners#x0027; Continuous Performance Test II (CPTII) (28); and the Kaufman Assessment Battery for Children II (KABC-II) Block Counting and Gestalt Closure subtests (29–30). Selected tests from this battery were grouped as probes of 4 cognitive domains: working memory (WISC IV Digit Span and Letter-Number Sequencing), psychomotor speed (WISC IV Coding and Symbol Search and Conners#x0027; CPTII), attention (Conners#x0027; CPTII and Inhibition and Color Naming score), and visuoconstructional ability (WASI Block Design and KABC-II Block Counting and Gestalt Closure).

Age-adjusted published norms were used to score participants#x0027; performance on each of the formal neuropsychological tests, with results expressed as Z scores with a mean of 0 and SD of 1 for a normative healthy population. The Z scores of the 2 tests defining a given cognitive domain (working memory, psychomotor speed, attention, and visuoconstructional ability) were averaged. The sum of the domain Z scores was used to estimate overall cognitive performance.

In the absence of a generally accepted definition of neurocognitive deficit (31), the following approach, based on previous work by our group and others (12,32), was used to categorize patients with childhood-onset SLE according to their cognitive ability into a group of patients with normal cognition and a group of patients with neurocognitive deficit. Based on the domain Z scores described above, the group of patients with childhood-onset SLE with normal cognition comprised patients who obtained, at most, 1 domain Z score of −1 or less, with their other domain scores greater than −1; the group of patients with neurocognitive deficit consisted of patients with 2 or more domain Z scores of −1 or less or 1 or more domain Z scores of −2 or less.

### MRI and image processing

Subjects recruited in Cincinnati underwent MR scanning on a Philips Achieva 3T scanner with an 8-channel head coil, while those recruited in Chicago were scanned on a Siemens Tim Trio 3T system with a 12-channel head coil. The T1 scans were acquired using a 3-dimensional magnetization-prepared rapid gradient-echo sequence with the following parameters for the Philips Achieva scanner: repetition time (TR) 2,300 msec, echo time (TE) 2.9 msec, inversion time (TI) 904.45 msec, flip angle 9°, slice thickness 1 mm, field of view 256 × 256 mm^2^, number of slices 176, and voxel size 1 × 1 × 1 mm^3^. The corresponding sequence parameters on the Siemens scanner were TR 2,300 msec, TE 3.13 msec, TI 900 msec, flip angle 8°, slice thickness 1 mm, rectangular field of view 240 × 256 mm^2^, number of slices 176, and voxel size 1 × 1 × 1 mm^3^.

Voxel-based morphometry was performed using the VBM8 toolbox (http://dbm.neuro.uni-jena.de/vbm), which is an extension to the SPM8 software (Wellcome Trust Centre for Neuroimaging). Briefly, images were corrected for bias-field inhomogeneities, registered to a standard template in MNI (Montreal Neurological Institute) space using both linear (12-parameter affine) and nonlinear transformations, and segmented into gray matter, white matter, and cerebrospinal fluid (34,35). Image normalization was further refined by high-dimensional warping using DARTEL (37) to the IXI550 template (http://www.brain-development.org). The tissue segments were additionally denoised using a spatially adaptive nonlocal means filter (38) and a hidden Markov random field model (35).

Gray matter and white matter tissue segments were modulated for the nonlinear component of normalization. This has the effect of scaling the voxel values such that the original amount of brain tissue is preserved after adjusting for individual brain sizes. As a consequence, brain size does not have to be included as a covariate in the statistical analysis (http://dbm.neuro.uni-jena.de/vbm/segmentation/modulation) (15–39). Finally, the images were smoothed with an 8-mm full-width half-maximum Gaussian kernel. Although the smoothing operation mildly reduces the spatial resolution of the images, it limits noise and makes the image voxel values more normally distributed (17).

### Statistical analysis

Statistical significance was based on cluster thresholds determined using the threshold-free cluster enhancement algorithm (40) together with nonparametric permutation-based testing (10,000 permutations for each analysis) as implemented in the “randomise” function of FSL (http://www.fmrib.ox.ac.uk/fsl/). Permutation testing is particularly useful for structural image analyses using cluster thresholds because it avoids overly liberal *P* values and Type I errors that can be seen with standard parametric statistics (41). All results were thresholded at *P* < 0.05 using a familywise error rate correction for multiple comparisons across the entire brain (unless otherwise indicated).

Two-sample *t*-tests were used for group comparisons, and the effects of scanning site, disease duration, and steroid use were discounted in the group comparisons using analysis of covariance (ANCOVA) models. Age and sex were not included as covariates since they did not differ between groups. Multiple regression analysis was used to examine the correlations between disease duration, steroid dose, and gray matter and white matter volumes. Analysis of demographic, neuropsychological, and other nonimaging variables was performed using IBM SPSS statistics (version 20). Post hoc tests were corrected for multiple comparisons using Tukey#x0027;s honestly significant difference test for demographic variables and Dunnett#x0027;s T3 test for neuropsychological variables (Z scores of the 4 cognitive domains and neurocognitive deficit status), since the latter did not assume equal variances between groups.

## RESULTS

### Study participants

There were 14 patients with childhood-onset SLE with normal cognition, and 8 with neurocognitive deficit. Three of the 22 controls scored in the range indicating neurocognitive deficit on neuropsychological testing and were excluded from the study. Neuropsychological testing was performed within 1 week of MR scanning in all but one instance, for a patient with childhood-onset SLE who was classified as having neurocognitive deficit. This particular subject had clinical neuropsychological testing 1 year prior to MR scanning, and interim neuropsychological assessments indicated the continued presence of neurocognitive deficit. This patient with neurocognitive deficit was excluded when calculating the neuropsychological test scores, and when regressing test scores against gray matter and white matter volumes (see Supplementary Figure 1, available on the *Arthritis & Rheumatism* web site at http://onlinelibrary.wiley.com/doi/10.1002/art.38009/abstract).

Controls and the patients with normal cognition were similar with regard to all sociodemographic variables (Table1). There were no significant differences between the patients with normal cognition and patients with neurocognitive deficit in either oral or intravenous corticosteroid use, either at the time of the study or cumulatively since diagnosis with childhood-onset SLE. Household income was lower in the neurocognitive deficit group than in the normal cognition group. There was a larger proportion of African American individuals in the neurocognitive deficit group than in the normal cognition or control groups (*P* < 0.05). Well-being was lower and both disease activity and depression (measured by the Children#x0027;s Depression Inventory #x005B;CDI#x005D;) were higher in the group with neurocognitive deficit than in the normal cognition group (*P* ≤ 0.05 for all). However, overall none of the subjects scored in the range indicating depression on the CDI.

**Table 1:** Demographic characteristics of the study participants*

	Controls (n = 19)	All patients with childhood-onset SLE (n = 22)	Patients with childhood-onset SLE with normal cognition (n= 14)	Patients with childhood-onset SLE with neurocognitive deficit (n=8)	*P*†
Age, years	14.3 ± 2.20	14.9 ± 2.01	14.7 ± 2.14	15.2 ± 1.82	NS
No. (%) female	15 (79)	18 (82)	11 (79)	7 (88)	NS
School grade	8.5 ± 2.37	8.8 ± 2.11	8.9 ± 2.35	8.6 ± 1.77	NS
Children#x0027;s Depression Inventory, T score‡	43.7 ± 5.9	43.9 ± 7.1	41.3 ± 3.8	48.4 ± 9.3	0.013
Estimated annual household income, US dollars	59,711 ± 45,423	66,409 ± 46,480	84,429 ± 48,889	34,875 ± 16,022	0.0028
Highest maternal education level, no.§					NS
High school	7	8	4	4	
Some college	7	7	4	3	
Bachelor#x0027;s degree	4	4	4	0	
Postgraduate education	1	2	2	0	
Race, no. (%)					0.028
White	9 (47)	9 (41)	8 (57)	1 (13)	
African American	9 (47)	11 (50)	4 (29)	7 (87)	
Biracial	1 (6)	2 (9)	2 (14)	0 (0)	
Ethnicity, no. (%) non-Hispanic	2 (11)	20 (91)	12 (86)	8 (100)	NS
Disease duration, months	NA	27.2 ± 24.0	29.5 ± 26.0	23.4 ± 21.1	NS
Medication					
Current daily dose of prednisone, mg/day	NA	16.84 ± 19.62	9.8 ± 8.75	29.1 ± 27.24	NS
Cumulative dose of oral corticosteroids, gm prednisone equivalent¶	NA	6.09 ± 5.75	5.23 ± 5.11	7.60 ± 2.41	NS
Cumulative dose of IV methylprednisolone, gm prednisone equivalent¶	NA	16.03 ± 41.87	19.13 ± 50.63	10.62 ± 21.13	NS
No. (%) treated with immunosuppressants	NA	10 (45)	5 (36)	5 (62.5)	NS
No. (%) treated with antihypertensive agents	NA	7 (32)	4 (29)	3 (37.5)	NS
Disease activity and damage					
SLEDAI-2K#	NA	5.82 ± 5.34	4.14 ± 2.98	8.75 ± 7.32	0.05
Physician assessment of disease activity**	NA	2.45 ± 2.09	2.35 ± 2.23	2.62 ± 1.92	NS
SDI score††	NA	0.45 ± 0.86	0.36 ± 0.74	0.63 ± 1.06	NS
Patient assessment of overall well-being	NA	7.86 ± 1.55	8.71 ± 1.07	6.38 ± 1.06	<0.0001

* Except where indicated otherwise, values are mean ± SD. There were no significant differences between controls and all patients with childhood-onset systemic lupus erythematosus (SLE). NS = not significant; NA = not applicable; IV = intravenous; SLEDAI-2K = SLE Disease Activity Index 2000; SDI = Systemic Lupus International Collaborating Clinics/American College of Rheumatology Damage Index.

† Patients with normal cognition versus patients with neurocognitive deficit, by *t*-test and chi-square or Fisher#x0027;s exact test, when appropriate.

‡ Range 34–100, where 34 indicates no depression.

§ Data on maternal education were missing for 1 patient with childhood-onset SLE with neurocognitive deficit.

¶ Cumulative steroid doses since diagnosis.

# Range 0–105, where 0 indicates inactive disease.

** Measured on a 0–10-cm visual analog scale, where 0 indicates inactive disease and 10 indicates very active disease.

†† Range 0–46, where 0 indicates no damage.

As expected, patients with neurocognitive deficit had significantly lower Z scores than both the controls and patients with normal cognition on neuropsychological tests probing working memory, psychomotor speed, and visuoconstructional ability, as well as an overall measure of cognition reflecting an average of the domain scores (all *P* < 0.005) (Table2). Six of the patients with neurocognitive deficit and 1 of the patients with normal cognition had a history of previous neuropsychiatric manifestations of NPSLE (*P* = 0.002). Specific symptoms at the time of disease onset in subjects classified as having childhood-onset SLE with neurocognitive deficit included headaches (n = 1), seizures (n = 2), and depression (n = 4). In the patient with normal cognition, the symptom history included depression, cognitive slowing, and difficulty focusing. These early NPSLE symptoms had all resolved by the time of the study.

**Table 2:** Study participants#x0027; performance on neuropsychological tests*

Neuropsychological performance by domain	Controls (n = 19)	Patients with childhood-onset SLE with normal cognition (n = 14)	Patients with childhood-onset SLE with neurocognitive deficit (n = 8)	*P*†	*P*‡
Overall cognition	−0.08 ± 0.50	0.13 ± 0.34	−0.93 ± 0.30	<0.0001	<0.0001
Working memory	−0.08 ± 0.68	−0.15 ± 0.52	−1.02 ± 0.54	0.0011	0.0036
Psychomotor speed	−0.14 ± 0.58	0.23 ± 0.69	−1.07 ± 0.53	0.0014	<0.0001
Attention	0.06 ± 0.55	0.13 ± 0.57	−0.36 ± 0.99	NS	NS
Visuoconstructional ability	−0.16 ± 0.90	0.30 ± 0.46	−1.28 ± 0.84	0.0019	<0.001

* Values are the mean ± SD Z score. There were no significant differences between controls and patients with childhood-onset systemic lupus erythematosus (SLE) with normal cognition. NS = not significant.

† Controls versus patients with childhood-onset SLE with neurocognitive deficit, by *t*-test.

‡ Patients with childhood-onset SLE with normal cognition versus patients with childhood-onset SLE with neurocognitive deficit, by *t*-test.

### Global changes in gray matter and white matter volumes

The global gray matter and white matter volumes and differences between groups are summarized in Table3. There were no differences in global gray matter or white matter volumes between controls and the patients with normal cognition. In contrast, the global gray matter volume in the group of patients with neurocognitive deficit was significantly smaller than that in the control group (*P* = 0.00082) and the group of patients with normal cognition (*P* = 0.0031). There was a trend toward lower white matter volumes in the group of patients with neurocognitive deficit when compared to either the control group (*P* = 0.061) or the group of patients with normal cognition (*P* = 0.065).

**Table 3:** Global gray matter and white matter volumes in the study participants*

	Gray matter	White matter
Controls (n =19)	600.7 ± 66.2	422.3 ± 39.7
All patients with childhood-onset SLE (n =22)	569.1 ± 101.9	406.8 ± 50.7
Patients with childhood-onset SLE with normal cognition (n =14)	597.4 ± 68.2	423.0 ± 42.7
Patients with childhood-onset SLE with neurocognitive deficit (n =8)	519.5 ± 40.4	378.3 ± 53.4
*P*	
Controls versus all patients with childhood-onset SLE	0.15	0.28
Controls versus patients with childhood-onset SLE with normal cognition	0.89	0.96
Controls versus patients with childhood-onset SLE with neurocognitive deficit	0.00082	0.061
Patients with childhood-onset SLE with normal cognition versus patients with childhood-onset SLE with neurocognitive deficit	0.0031	0.065

* Values are the mean ± SD cm^3^. SLE = systemic lupus erythematosus.

### Decreased regional gray matter volume in patients with neurocognitive deficit

**Figure 1:**
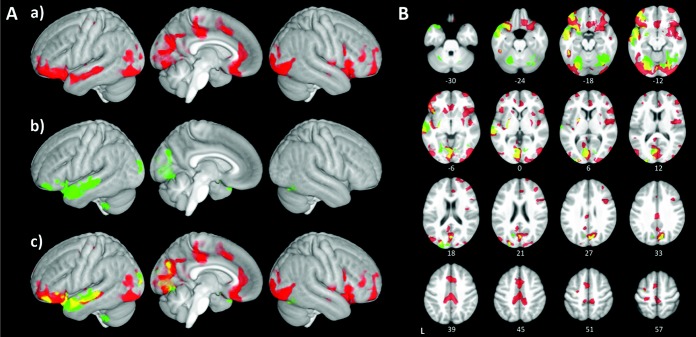
Decreases in gray matter in patients with childhood-onset systemic lupus erythematosus (SLE) with neurocognitive deficit versus controls and patients with childhood-onset SLE with normal cognition. Red, green, and yellow designate differences in gray matter volume that were statistically significant at *P* < 0.05 after correction for multiple comparisons across the entire brain. A, Sagittal surface 3-dimensional views of a, regions with decreased gray matter in patients with neurocognitive deficit versus controls (red), b, regions with decreased gray matter in patients with neurocognitive deficit versus patients with normal cognition (green), and c, overlap (yellow) between the comparisons shown in a and b. Compared to the controls and patients with childhood-onset SLE with normal cognition, the patients with SLE with neurocognitive deficit showed extensive areas of decreased gray matter in the limbic cortex (orbitofrontal and cingulate), inferior frontal, temporal, and visual association cortex. B, Axial sections showing details of regions with gray matter decreases in patients with childhood-onset SLE with neurocognitive deficit. Differences between the controls and patients with childhood-onset SLE with neurocognitive deficit are shown in red; differences between the patients with childhood-onset SLE with normal cognition and those with neurocognitive deficit are shown in green. Overlapping areas of decreases in gray matter volume are shown in yellow. Numbers indicate normalized (in Montreal Neurological Institute space) slice locations in millimeters in the z direction.

There was extensive gray matter volume loss in the patients with neurocognitive deficit compared to controls and patients with normal cognition (Figure 1), especially in the lateral frontal, orbitofrontal, anterior cingulate, and lateral temporal areas as well as visual association regions. Locations and statistics of the clusters that significantly differed between groups are provided in Supplementary Tables 1 and 2, available on the *Arthritis & Rheumatism* web site at http://onlinelibrary.wiley.com/doi/10.1002/art.38009/abstract. We found neither duration of childhood-onset SLE nor cumulative (oral or intravenous) steroid exposure to be associated with the gray matter volume alterations observed in the patients with neurocognitive deficit (for details see Supplementary Figure 2, available on the *Arthritis & Rheumatism* web site at http://onlinelibrary.wiley.com/doi/10.1002/art.38009/abstract).

### Decreased regional white matter volume in patients with neurocognitive deficit

**Figure 2:**
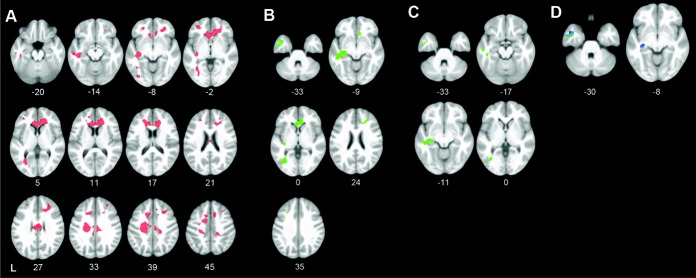
Differences in white matter volume between controls, patients with childhood-onset systemic lupus erythematosus (SLE) with normal cognition, and patients with childhood-onset SLE with neurocognitive deficit. A, Regions showing a trend toward decreased white matter volume in patients with childhood-onset SLE with neurocognitive deficit versus controls (0.05 < corrected *P* ≤ 0.1) (red). There were no regions showing a significant difference in white matter volume between controls and patients with childhood-onset SLE with neurocognitive deficit at *P* < 0.05. B, Decreased white matter volume in patients with childhood-onset SLE with neurocognitive deficit compared with patients with childhood-onset SLE with normal cognition (corrected *P* < 0.05) in the left temporal lobe, anterior corpus callosum, and bilateral dorsal frontal white matter (green). C, Persistence of decreases in white matter volume in the left temporal lobe (corrected *P* < 0.05) (green) but not in the anterior callosum or dorsal frontal white matter in patients with SLE with neurocognitive deficit after correction for differences in disease duration between patients with neurocognitive deficit and patients with normal cognition (by analysis of covariance #x005B;ANCOVA#x005D;). D, Trends toward decreased white matter volume in patients with childhood-onset SLE with neurocognitive deficit (0.05 < corrected *P* ≤ 0.1) (blue/green) after correction for differences in oral or intravenous steroid use between the patients with neurocognitive deficit and those with normal cognition (by ANCOVA). Numbers indicate normalized (in Montreal Neurological Institute space) slice locations in millimeters in the z direction.

As shown in Figure 2A, there was a trend toward decreased white matter volumes in several brain regions in patients with neurocognitive deficit compared to controls (0.05 < *P* ≤ 0.1). Conversely, differences in white matter volumes between the patients with neurocognitive deficit and the patients with normal cognition reached statistical significance in the anterior corpus callosum, left temporal lobe, dorsal frontal corona radiata, and frontal pole (Figure 2B).

After adjustment for disease duration (by ANCOVA), several regions in the left temporal lobe still showed significantly reduced white matter volumes in the patients with neurocognitive deficit versus the patients with normal cognition (Figure 2C). Similarly, after accounting for cumulative (oral and intravenous) steroid exposure (by ANCOVA), there remained only a trend toward decreased white matter volume in the left temporal lobe in patients with neurocognitive deficits compared to patients with normal cognition (Figure 2D).

### Differential associations between oral versus intravenous pulse steroids and white matter volumes in childhood-onset SLE

**Figure 3:**
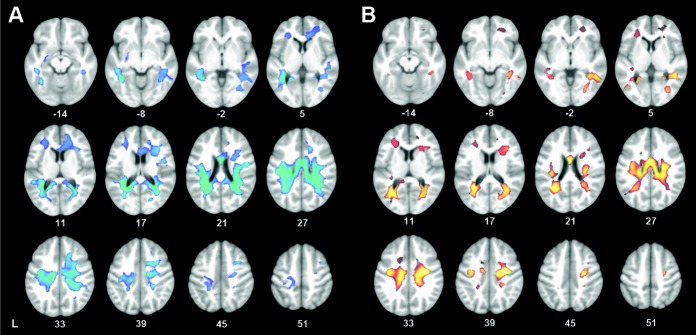
A, Multiple regression across all patients with childhood-onset systemic lupus erythematosus (SLE), showing a negative relationship between white matter volume and cumulative oral steroid use (corrected *P* < 0.05) (blue/green) after controlling for disease duration and cumulative intravenous steroid use. B, Multiple regression across all patients with childhood-onset SLE, showing a positive relationship between white matter volume and cumulative intravenous steroid use (corrected *P* < 0.05) (red/yellow) after controlling for disease duration and cumulative oral steroid use. Numbers indicate normalized (in Montreal Neurological Institute space) slice locations in millimeters in the z direction.

Interestingly, there was a differential association between oral versus intravenous pulse steroids and white matter volumes in both childhood-onset SLE groups (those with normal cognition and those with neurocognitive deficit). White matter volumes throughout the cerebral hemispheres showed a negative association with the cumulative dose of oral steroids (Figure 3A) but a positive association with the cumulative exposure to intravenous steroids (Figure 3B).

## DISCUSSION

This study is the first to use voxel-based morphometry, an innovative, quantitative MRI-based approach to the measurement of gray matter and white matter volumes, to demonstrate that childhood-onset SLE with neurocognitive deficit is associated with decreased gray matter and white matter volumes, while gray matter and white matter volumes in children with childhood-onset SLE with normal cognition are comparable to those in healthy children. The results of our study are consistent with similar observations in adults with SLE. Using high-resolution structural MRI and advanced image processing techniques, previous studies have shown that adult patients with SLE-associated neurocognitive deficit have smaller gray matter and/or white matter volumes than SLE patients with normal cognition (14,15). Our study provides new strong quantitative evidence that SLE is associated with similar morphologic alterations in the developing brains of children with childhood-onset SLE–associated neurocognitive deficit.

Likewise, our results support the findings of a previous study that demonstrated global cerebral volume loss in untreated patients with childhood-onset SLE compared with controls, using qualitative rater-dependent measures of cerebral atrophy (12). Patients included in that study underwent brain imaging as part of clinical care; hence, they had clinical features compatible with neurologic compromise, although the results of formal neuropsychological testing were not available.

In the present study, clinically overt neurocognitive deficit was associated with global gray matter atrophy and marked regional declines in gray matter and white matter volumes. Similar global (12) and multifocal regional areas of gray matter volume loss in the frontal, temporal, and occipital cortex have been reported in previous studies of adult patients with diverse NPSLE syndromes (14–16). For example, Cagnoli et al (15) described, in comparison to healthy controls, gray matter volume loss in the frontal and parietal regions and thalamus in adult SLE patients with and those without NPSLE.

The cumulative effects of NPSLE and childhood-onset SLE with neurocognitive deficit on brain volumes are unknown. Given the chronic and fluctuating course of childhood-onset SLE in other organ systems, it may be expected that the children with childhood-onset SLE with neurocognitive deficit included in our study more often had a prior history of NPSLE than did childhood-onset SLE patients with normal cognition. Our previous functional imaging studies also suggest that even in patients in whom clinical features of NPSLE have resolved completely, altered neuronal network activity can persist (42). However, the cross-sectional nature of the present study does not allow us to differentiate whether the decreases in gray matter and white matter represent an acute change related to the patient#x0027;s current neurocognitive status or if brain volume changes could be related to the cumulative insult of repeated central nervous system involvement. Future longitudinal studies of children with childhood-onset SLE will be necessary to distinguish these possibilities.

The relationship between brain atrophy and disease duration in SLE remains a subject of controversy (9–44). Although a previous study of adult SLE showed decreased gray matter volume to be associated with longer disease durations (14), we found disease duration to be associated only with decreased white matter, but not gray matter, volumes in childhood-onset SLE. One possible explanation for this discrepancy could be the shorter disease durations of the patients with childhood-onset SLE included in our study as compared to much longer disease durations of participants in studies of adult SLE (14). Furthermore, Muscal et al (12) also failed to find an association between disease duration and global brain atrophy in children with childhood-onset SLE. Whether there are differences between children and adults in the underlying inflammatory processes, brain plasticity, or other compensatory brain processes remains to be determined.

Systemic steroids are the cornerstone for the treatment of moderate and major organ involvement in SLE and childhood-onset SLE. In this study we confirmed in childhood-onset SLE observations previously made in adults with SLE, that steroid exposure is associated with changes in brain volumes (9,43). However, we also showed for the first time that oral steroid use appears to be associated with significant decreases in white matter but not gray matter volumes, while greater intravenous pulse steroid exposure is positively correlated with greater regional white matter volumes in childhood-onset SLE. Although the reasons for these observations are unclear, one might hypothesize that this could represent differences in the effects of pulse as compared to oral corticosteroids on the cytokine profile in SLE, with only pulse corticosteroids eliminating the characteristic interferon-α signature (46). If confirmed in larger and preferably longitudinal studies, this finding might prove relevant for the treatment of NPSLE. The observation that certain therapies may reduce or even prevent white matter volume loss is also consistent with a report by Xu et al, who found that treatment with immunosuppressants was associated with preserved white matter volumes in adults with SLE (47).

It is tempting to relate decreased gray matter volume in childhood-onset SLE with neurocognitive deficit to the particular cognitive alterations seen in this group and more generally to patients with NPSLE. However, the actual relationships between regional gray matter and white matter volume changes and either the behavioral or the neurocognitive deficits are likely to be highly complex. Volume changes may also represent a rather downstream effect. For example, we found visuoconstructional performance to be associated with gray matter volume loss in the orbitofrontal, dorsolateral prefrontal, and anterior cingulate cortex (see Supplementary Figure 1, available on the *Arthritis & Rheumatism* web site at http://onlinelibrary.wiley.com/doi/10.1002/art.38009/abstract). Although these regions are not commonly thought to affect visuoconstructional abilities, the standardized cognitive tests used for the visuoconstructional ability domain (WASI Block Design and KABC-II Block Counting) also require significant executive and motor contributions and this may relate to the association with frontal lobe involvement. Thus, further work is needed both longitudinally and across the spectrum of cognitive deficits to further our understanding of the relationships between volumetric changes and neuropsychological performance.

An important methodological feature of the present study is the use of nonparametric permutation-based statistics. This type of analysis avoids assumptions about the statistical properties of the images (41). Through the use of permutation-based statistics and automatic cluster thresholding (40), we were better able to detect group differences in gray matter and white matter in the present study (improved sensitivity) while still properly controlling for multiple comparisons (improved specificity).

Our study also needs to be viewed in the light of certain limitations. Although the patients with childhood- onset SLE with neurocognitive deficit were similar to controls with regard to measures of race, ethnicity, and estimated annual family household income, i.e., a proxy measure for socioeconomic status, we were unable to match the patients with normal cognition and patients with neurocognitive deficit for all potentially relevant sociodemographic factors or depression. Because of the correlation of these measures with each childhood-onset SLE group, we are unable to distinguish the role these factors may be playing in the changes seen in gray matter and white matter volumes (48–49). The present study does not allow us to assess whether increased depression in the neurocognitive deficit group reflected the patients#x0027; disease state, socioeconomic status, or other factors, or if depression contributed to changes in brain structure. Thus, it will be important in future studies to distinguish direct disease-related effects on brain tissue from indirect effects such as medications, including corticosteroids and other immunosuppressants, or from patient features, such as socioeconomic status.

Another potential limitation of the present study, and indeed of other studies of childhood-onset SLE, is the lack of a consistent, widely accepted definition for identifying patients with neurocognitive deficit (31). This study used criteria similar to what we and others have previously employed (11,32). Nevertheless, the variability of these criteria for neurocognitive deficit and the lack of clinical differences in some studies of healthy controls and children with childhood-onset SLE stress the importance of developing validated criteria for childhood-onset SLE–associated neurocognitive deficit and defining the most appropriate choice of control populations (31–50).

The etiology of neurocognitive deficit is manifold, and we excluded the very small number of control subjects with neurocognitive deficit (n = 3) from our study since we were focused on examining differences between healthy controls, patients with childhood-onset SLE–associated neurocognitive deficit, and patients with childhood-onset SLE with normal cognition. Thus, future studies are warranted to delineate whether childhood-onset SLE–associated neurocognitive deficit differs from other forms of pediatric neurocognitive deficit with respect to gray matter and white matter changes.

In summary, this study is the first to show gray matter and white matter volume loss in children with childhood-onset SLE in whom formal neurocognitive testing suggests abnormally low cognition. Conversely, brain volumes of childhood-onset SLE patients with normal cognition were found to be no different from those of healthy children with normal cognition. In contrast to some adult studies, we did not find disease duration to be associated with gray matter volume changes. We also show for the first time that intravenous pulse steroids, but not oral steroids, may be related to preserved white matter volumes. The relationship between brain volumes and clinical features in childhood-onset SLE raises the expectation that morphometric parameters may be well suited to serve as outcome measures in studies focusing on the etiology and treatment of NPSLE.

## AUTHOR CONTRIBUTIONS

All authors were involved in drafting the article or revising it critically for important intellectual content, and all authors approved the final version to be published. Dr. Gitelman had full access to all of the data in the study and takes responsibility for the integrity of the data and the accuracy of the data analysis.

**Study conception and design.** Gitelman, Klein-Gitelman, Ying, Zelko, DiFrancesco, Brunner.

**Acquisition of data.** Klein-Gitelman, Sagcal-Gironella, Zelko, Beebe, DiFrancesco, Parrish, Hummel, Brunner.

**Analysis and interpretation of data.** Gitelman, Ying, Zelko, DiFrancesco, Beckwith, Brunner.
