# Supplementary material for: Brain Morphometric Changes Associated With Childhood-Onset Systemic Lupus Erythematosus and Neurocognitive Deficit
Source: Arthritis Rheum. 2013 Jul 26;65(8):2190–200. doi: 10.1002/art.38009 (PMC3840703; doi:10.1002/art.38009)
Supplement: Supplementary file 1 [file art0065-2190-sd1.docx]

**SUPPLEMENTARY MATERIAL**


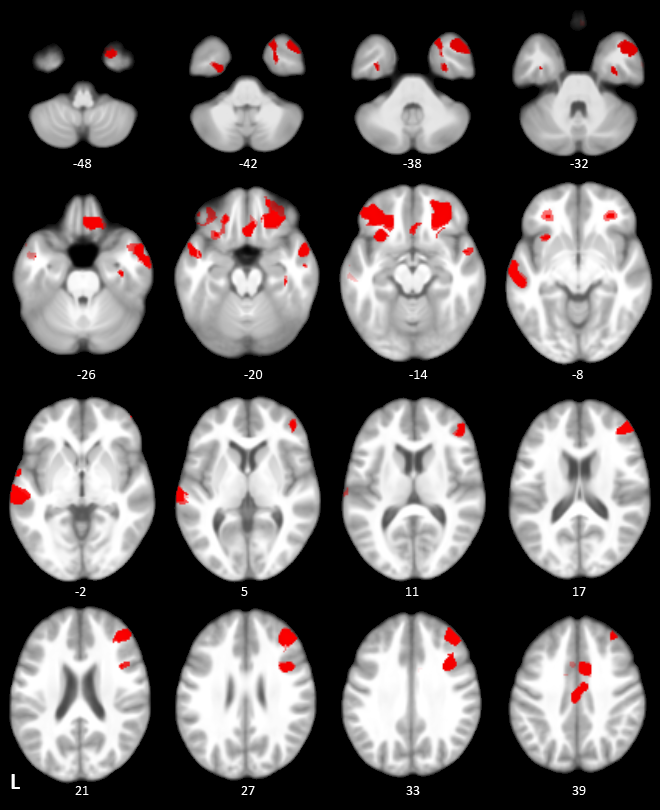


**Supplementary Figure 1.** Multiple regression VCA scores vs. GM volume: Red areas are clusters showing reduced gray matter volume across all cSLE subjects that correlated with lower Z scores for the VCA domain.
