# Supplementary material for: Brain Morphometric Changes Associated With Childhood-Onset Systemic Lupus Erythematosus and Neurocognitive Deficit
Source: Arthritis Rheum. 2013 Jul 26;65(8):2190–200. doi: 10.1002/art.38009 (PMC3840703; doi:10.1002/art.38009)
Supplement: Supplementary file 2 [file art0065-2190-sd2.docx]

**SUPPLEMENTARY MATERIAL**

**
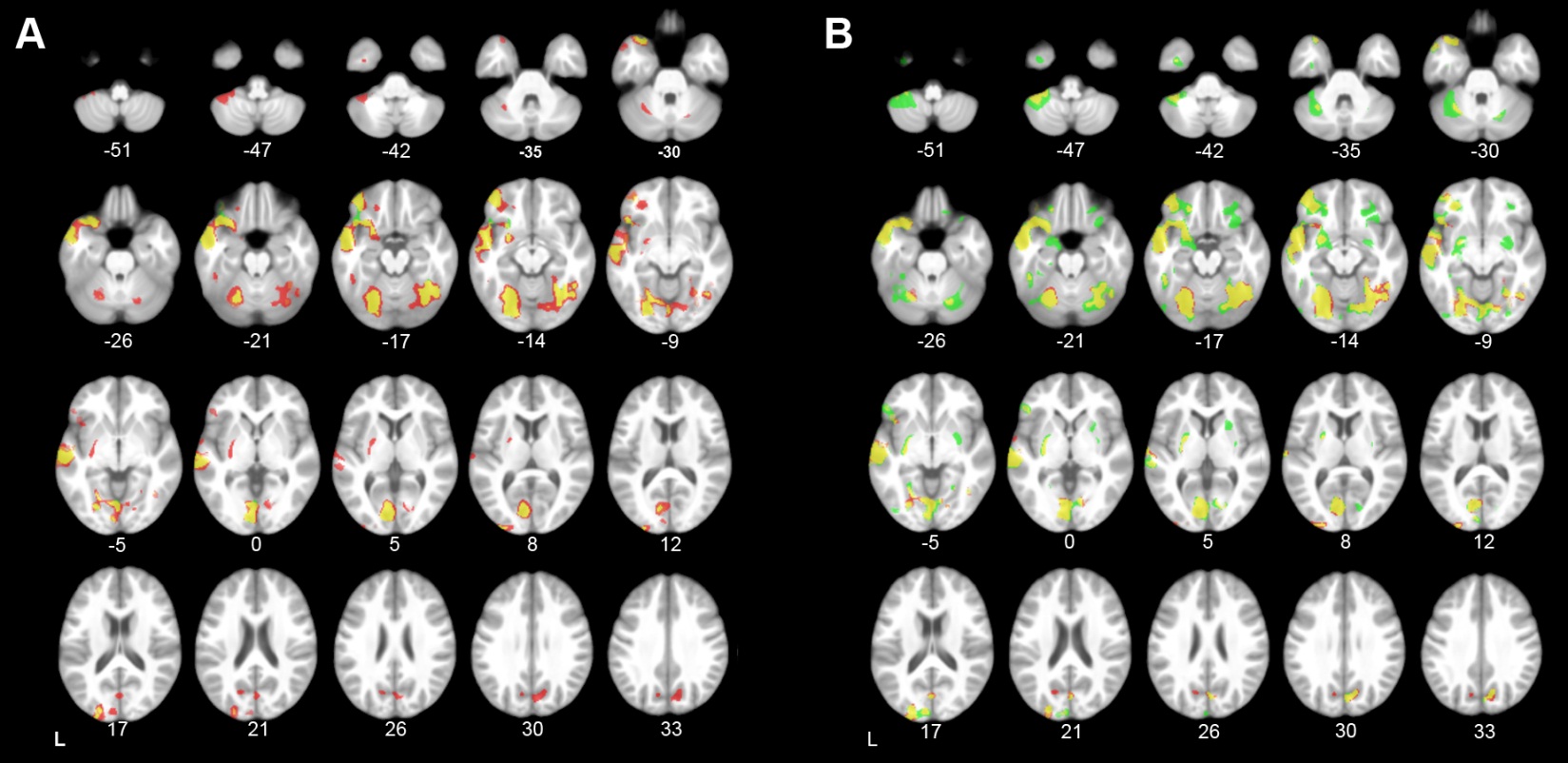
**

**Supplementary Figure 2**: **Panel A** shows regions with decreased GM volume in the cSLE-NCD vs. cSLE-NL comparison for ANCOVA models that did (green) or did not (red) include SLE duration as a covariate. Voxels where GM decreases for both models overlapped are colored yellow. The figure shows a high degree of overlap between the two models consistent with there being no overall association of GM volume decreases with disease duration. The lack of association between cumulative oral or IV steroid exposure and GM volume is demonstrated in **Panel B**, with decreased GM volume in the cSLE-NCD vs. cSLE-NL comparison for models that did (green) or did not (red) include cumulative oral and IV steroid doses as covariates. Again the large extent of overlap between these models (yellow) is consistent with differences in steroid exposure not being associated with decreased GM volume in the cSLE-NCD group. Note that the increased number of green voxels representing the model that included steroid dose as a covariate, suggests this model better accounted for overall non-specific variance even though the cumulative steroid dose (oral or IV) did not explain the reduction in gray matter volume when tested using multiple regression.
