# Supplementary material for: Brain Morphometric Changes Associated With Childhood-Onset Systemic Lupus Erythematosus and Neurocognitive Deficit
Source: Arthritis Rheum. 2013 Jul 26;65(8):2190–200. doi: 10.1002/art.38009 (PMC3840703; doi:10.1002/art.38009)
Supplement: Supplementary file 3 [file art0065-2190-sd3.docx]

**SUPPLEMENTARY MATERIAL**

**Supplementary Table 1: Controls vs. cSLE-NCD cluster statistics**

| Cluster size (vox) | Max Z‑score | X (cog)  mm | Y (cog)  mm | Z (cog)  mm | Region(s) |
| --- | --- | --- | --- | --- | --- |
| 14637 | 6.14 | -3.5 | -77.5 | -0.5 | Left lingual gyrus, fusiform gyrus, lateral occipital, calcarine inferior temporal, posterior cingulate; midline cuneus; Right lingual, parieto-occipital, precuneus, fusiform, occipital pole, lateral occipital |
| 11450 | 4.5 | 30.2 | 19.9 | -5.7 | Right insula: frontal pole, orbitofrontal, precentral gyrus, , inferior frontal gyrus, middle frontal gyrus, superior temporal gyrus, putamen, pallidum; Left subgenual cingulate, caudate |
| 8531 | 5.54 | -41.5 | 15.7 | -11.7 | Left orbitofrontal cortex, inferior frontal gyrus, superior temporal gyrus, middle temporal gyrus, insula |
| 3058 | 4.46 | 0.7 | -30.2 | 44.5 | Posterior cingulate, Right precuneus, paracentral lobule |
| 1520 | 4.89 | -0.5 | 9.4 | 46 | Left anterior cingulate, bilateral supplementary motor area. |
| 597 | 4.3 | 43.3 | 39.8 | 15.6 | Right middle frontal gyrus, inferior frontal gyrus. |
| 413 | 3.95 | 24 | -90.4 | 18.6 | Right occipital pole |
| 360 | 4.08 | -22.4 | -88.3 | 20.2 | Left occipital pole |
| 278 | 4.68 | -24.8 | -29.1 | 58.3 | Left postcentral gyrus |
| 171 | 3.84 | -25 | -97.7 | 7.9 | Left occipital pole |
| 122 | 4.05 | -27.7 | -.10.5 | 53.6 | Left precentral gyrus |
| 84 | 3.57 | -11.4 | 43.6 | 0.5 | Left anterior cingulate gyrus |
| 35 | 3.04 | -50.7 | -42.8 | 10.1 | Left superior temporal gyrus |
| 28 | 3.6 | -33.7 | 38.4 | 11.6 | Left frontal pole |
| 15 | 3.99 | -23.5 | -17.7 | 67.8 | Left precentral gyrus |
| 12 | 3.03 | 12.6 | 3.1 | 37.6 | Right anterior cingulate gyrus. |

The table lists all significant clusters for the specified contrast. Cluster size is in voxels (vox). Max z-score is for the peak value in the cluster. XYZ locations are the center-of-gravity (COG) or center-of-mass of the cluster, which was not necessarily at the same location as the peak voxel. The listed regions under each cluster include all sub-peaks at least 8 mm apart within the cluster. Lobar affiliations are as follows: occipital cortex (fusiform, lingual, lateral occipital, calcarine, cuneus and occipital pole), temporal cortex (inferior, middle and superior temporal gyri), frontal cortex (inferior and middle frontal gyri, frontal pole, precentral gyrus, paracentral lobule, supplementary motor cortex and orbitofrontal cortex), and parietal cortex (precuneus, posterior cingulate).
