# Supplementary material for: Brain Morphometric Changes Associated With Childhood-Onset Systemic Lupus Erythematosus and Neurocognitive Deficit
Source: Arthritis Rheum. 2013 Jul 26;65(8):2190–200. doi: 10.1002/art.38009 (PMC3840703; doi:10.1002/art.38009)
Supplement: Supplementary file 4 [file art0065-2190-sd4.docx]

**SUPPLEMENTARY MATERIAL**

**Supplementary Table 2: cSLE-NL vs. cSLE-NCD cluster statistics**

| Cluster size (vox) | Max Z‑score | X (cog)  mm | Y (cog)  mm | Z (cog)  mm | Region(s) |
| --- | --- | --- | --- | --- | --- |
| 8102 | 5.72 | 1.7 | -69.2 | -9.3 | Right fusiform gyrus, lingual gyrus, calcarine fissure, lateral occipital cortex, inferior temporal cortex, parieto-occipital fissure, cerebellum V1 |
| 5378 | 6.03 | -6 | -47.2 | -1 | Left anterior superior temporal cortex, temporal pole, middle temporal gyrus, posterior putamen, insula, orbitofrontal cortex, amygdala |
| 864 | 4.61 | -37 | 42 | -13.1 | Left frontal pole, orbitofrontal cortex |
| 642 | 4.76 | -20.5 | -94 | -14.6 | Left occipital pole |
| 332 | 4.35 | -35 | -44.6 | -46.3 | Left cerebellum crus II, VIIIa |
| 118 | 3.3 | -13.3 | -71.2 | 25.2 | Left precuneus, parieto-occipital fissure |
| 115 | 4.76 | -46 | -43.2 | -19.2 | Left inferior temporal cortex |
| 82 | 3.24 | -19.7 | 34.3 | 18.3 | Left orbitofrontal cortex |
| 69 | 3.33 | -48.1 | 30.3 | -2.7 | Left inferior frontal cortex |
| 34 | 4.84 | -34.6 | -5.12 | -40.7 | Left inferior temporal |
| 24 | 5.5 | -30.1 | -12.9 | 54.1 | Left precentral gyrus |
| 2 | 3.38 | -21.8 | 23.3 | -15 | Left orbitofrontal |

See legend for supplementary table 1.
